# Investigating the Water State in Saccharide Solutions by Infrared/Far-Infrared Spectra in the 1000–100 cm^–1^ Region Combined with Bands in the 4000–3000 cm^–1^ Region

**DOI:** 10.1021/acs.jpca.4c08369

**Published:** 2025-06-10

**Authors:** Daitaro Ishikawa, Natsumi Shichishima, Rin Shinohara, Jiamin Yang, Tomoyuki Fujii

**Affiliations:** Graduate School of Agricultural Science, 13101 Tohoku University, 468-1, Aramaki Aza Aoba, Aoba-ku, Sendai, Miyagi 980-8572, Japan

## Abstract

Water molecules are arranged as tetrahedral structures, owing to the hydrogen-bonding network in solution. However, many aspects of their actual state remain unclear. This study analyzed saccharide solutions of various concentrations by infrared/far-infrared (IR/FIR) spectra in the 1000–100 cm^–1^ region and IR spectra in the 4000–3000 cm^–1^ region. The relationship between the second-derivative intensities of bands at 3210 and 3374 cm^–1^ attributed to strong and weak hydrogen bonds, for glucose, maltose, sucrose, and NaCl solutions at different concentrations, was described by the same straight line. Two bands were also extracted from the IR/FIR region, which are attributed to the ordered and distorted structures of the water. The ratio indices A_high_/A_low_ and A_3210_/A_3374_ were developed using the intensities of the bands at 673 and 403 cm^–1^ and at 3210 and 3374 cm^–1^, respectively. Both indices increased with the saccharide concentration. The results show that saccharide molecules contributed to the strengthening of the water structure. Moreover, the change in A_high_/A_low_ was small compared to that in A_3210_/A_3374_ at high saccharide concentrations. Therefore, a difference in the state of water possibly exists between those expressed by IR and IR/FIR spectra, and the complementary use of these spectra is effective in clarifying the state of water in solution.

## Graphical abstract

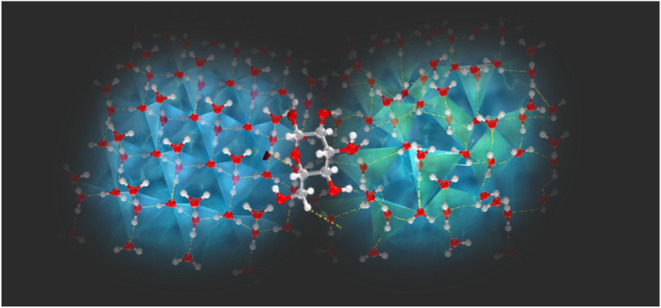

## Introduction

Water, the most abundant component on Earth, has a simple chemical structure. When in liquid form, water molecules are arranged as tetrahedral structures due to the formation of hydrogen bonds between them.^1–5^ While such tetrahedral structures of water molecules exist in aqueous solutions, their dynamics in such solutions are still largely unknown owing to the thermal fluctuations that are characteristic of liquids. Particularly, a conflict exists between mixture models—based on the distributions of structural components, reflecting the coexistence of two or more types of local structures—and continuum models—based on the distribution of continuous hydrogen networks.^6,7^ The mixture model proposed by Bernal and Fowler is a simple model in which water is a mixture of two molecular states.^6^ One is a low-density, low-energy state similar to that of ice, making it highly structured. The other is a high-density, high-energy state, wherein the molecules behave like an ordinary liquid. Water molecules are considered to be distributed between these two states in equilibrium. Subsequently, numerous theories on water have been proposed, many of which follow this mixture model.^6^ In these theories, the ice-like components are considered the hydrogen-bonded or structural components, whereas the other components are considered non-hydrogen-bonded or nonstructural components. In contrast, Pople considered that the imperfections that occur in the ice-like structure of water were due to strain rather than hydrogen bond breakage, and accordingly developed a continuum model for water.^7^ This model is characterized by the contribution from the second- and third-nearest-neighbor molecules being spread over a wider range than those from the nearest-neighbor molecules. This indicates that a variety of local-ordered structures of water are significantly larger in number than the ice-like structures. Overall, the continuum model describes the hydrogen-bonded network of water as a fully hydrogen-bonded (tetrahedrally coordinated) motif without any breakage. However, notably, Tanaka et al. have recently analyzed the X-ray scattering data of water in detail and demonstrated that two peaks exist within a diffraction peak.^8^ One of the hidden peaks was associated with the tetrahedral structure of water, but the other peak was found to arise from a distorted tetrahedral structure. This finding unambiguously proves the coexistence of the two states of local structures in liquid water, directly evidencing the two-state model.

The potential of the two-state model for water can also be explained by spectroscopic studies. Vibrational spectroscopy is one of the most sensitive methods for investigating the hydrogen-bonded structure in solutions.^9–15^ Analyzing the infrared (IR) spectra in the 4000–3000 cm^–1^ region for water and aqueous solutions revealed two major bands originating from O–H stretching vibration modes. The band at 3210 cm^–1^ was attributed to the O–H stretching mode of strongly hydrogen-bonded water species, whereas that at 3374 cm^–1^ was assigned to weakly hydrogen-bonded water species.^16^

The component of the O–H stretching band of water, centered around 3200 cm^–1^, is highly polarized and diminished at a perpendicular position.^17^ This band is known as the “collective band” and is ascribed to the water molecules that exhibit ν_1_ vibrations in phase with each other.^9,10^ Each vibration observed by this band is localized; therefore, the band is useful for evaluating the ordered structure of water from a local perspective. Interestingly, in the polarized Raman spectra of aqueous solutions of salt, the intensity of the collective band decreases with increasing salt concentration,^9,10^ indicating that the intensity of the band decreases with a decrease in the symmetry of the hydrogen network caused by the addition of salt. In particular, if the number of distorted hydrogen bonds increases, then the polarization is diminished; the intensity of the collective band likely reflects the degree of distortion. Therefore, Raman spectra in the 4000–3000 cm^–1^ region may be interpreted using the two-state model for water, with one state in which all water molecules form hydrogen bonds with ice-like regularity, and another state with distorted hydrogen bonding.

Moreover, bands arising from the intermolecular motion of water exist below 1000 cm^–1^; the band arising from libration motion is located in the 1000–400 cm^–1^ region; and the band originating from hindered translation motion is located below 300 cm^–1^.^18^ Ashihara et al. suggested that the librational L2 band, which has a maxima at 670 cm^–1^, is sensitive to water structure and can shift to other wavenumber values in the region 1000–100 cm^–1^.^19^ Cho et al. mainly attributed this band to intermolecular vibration, involving the bonding and collective vibration of several water molecules.^20^ Meanwhile, both Ashihara et al. and Cho et al. reported that the band in the 1000–100 cm^–1^ region arises from the cooperative vibrational mode of several to several hundred water molecules;^19,20^ this cooperative mode reflects structured water. While IR spectra are concerned with local vibrational modes, the 1000–100 cm^–1^ region is for the cooperative vibrational mode of numerous water molecules. Therefore, the 1000–100 cm^–1^ enables the evaluation of water states in more macro regions.

Ishikawa et al. reported that the position of the band with the highest intensity in the 1000–100 cm^–1^ region of LiCl, KCl, NaCl, CsCl, CaCl_2_, and BaCl_2_ solutions changed with increasing salt concentration.^21^ They also found that isosbestic points occurred in the 1000–100 cm^–1^ region and that their wavenumbers corresponded to the hydrated area.^21^ These results have scientific value for experimentally proving the two-state model of liquid water and clearly demonstrate that spectra in the 1000–100 cm^–1^ region can be a potential new method for evaluating objects containing water as a component. This study suggests that the simultaneous use of spectral regions with different characteristics can help to clarify the state of water. In recent years, indeed, Begušić and Blake have presented an attractive study on the evaluation of water structure using terahertz and IR spectra based on MD simulations.^22^ Nevertheless, no studies have focused on the 1000–100 cm^–1^ region, owing to which the discussion on the spectral behavior in this region is insufficient.

Carbohydrates are one of the most fascinating additives to water owing to the presence of hydroxyl groups in them, which affect the structure of water when they are dissolved. In fact, Hossain et al. reported that, in sugar-added solutions, the sugar gets incorporated into the structure of the water, thereby strengthening the water structure.^23^ Therefore, if evaluating the water structure of sugar-added solutions is possible from the ordered and distorted hydrogen-bonding perspectives, then the contribution of sugar to the structure of water can be clarified, and the usefulness of the spectra in the 1000–100 cm^–1^ region can be demonstrated for an intrinsic understanding of the state of water. Thus, in this study, to clarify the effects of added saccharides on the water structure, we performed evaluations using monosaccharides and disaccharides, which are simple hydrophilic molecules that can act as convenient models for more complex systems, such as biomaterials, to provide essential insights into the properties of aqueous media. To the best of our knowledge, this study is the first to evaluate the state of water in saccharide solutions based on experimental data through the complementary use of the spectra in the 1000–100 cm^–1^ and 4000–3000 cm^–1^ regions. Furthermore, we succeeded in identifying the difference in the correspondence between these two spectral regions for the water structure.

## Experimental Section

### Sample Preparation

Glucose, sucrose, and maltose (Wako Pure Chemical Industries, Ltd., Osaka, Japan) were dissolved in Milli-Q water to prepare 10–30% w/w solutions. NaCl was dissolved in Milli-Q water to prepare 0–22% w/w sample solutions, at intervals of 4% w/w; the NaCl solutions were prepared based on the method described by Ishikawa et al.^21^

### IR and IR/FIR Spectra in the 4000–100 cm^–1^ Region

IR and IR/FIR spectra in the 4000–100 cm^–1^ region were measured using an FT/IR6300 spectrometer (JASCO Co., Tokyo, Japan). The spectral measurements were performed at 25 °C and under 1 atm of pressure. The spectrometer was equipped with a broadband mid-IR and FIR replacement beam splitter (MFBB-6000BS, JASCO Co., Tokyo, Japan) and a deuterated l-alanine triglycine sulfate detector. The optical configuration of this spectrometer was appropriately designed to measure spectra below 650 cm^–1^, and the IR instrument was capable of simultaneously scanning over the entire wavenumber range of 4000–100 cm^–1^. The attenuated total reflection (ATR) method with a single reflection mode was applied for which an ATR unit (ATR-ProOne; JASCO, Tokyo, Japan) equipped with a diamond prism was used. The spectra were recorded at a spectral resolution of 4 cm^–1^, and 256 scans were conducted for each spectrum. The spectra were then analyzed using Spectral Manager software (JASCO Co., Tokyo, Japan), and an ATR correction was applied to correct the dependence of the penetration depth of the evanescent waves on the wavenumber. To identify the specific bands and correct the baseline drift, the spectra in the 4000–1000 cm^–1^ region were subjected to a second-derivative procedure, including Savitzky–Golay smoothing (second order and 41 points).^24^

## Results

### Comparing the Spectral Behaviors of the Collective Band and IR/FIR Spectra in the 1000–100 cm^–1^ Region for Saccharides and NaCl Solutions

The IR spectra in the 4000–3000 cm^–1^ region for the NaCl and saccharide solutions, as well as their second-derivative spectra, are shown in Figure 1. For both types of solutions, two bands existed at 3210 and 3374 cm^–1^ (indicating a defect or distorted hydrogen network of molecules).^17^ For the NaCl solutions, as the NaCl concentration increased, the intensity of the collective band decreased, and that of the band at 3374 cm^–1^ increased. Moreover, an isosbestic band appeared around 3300 cm^–1^. Meanwhile, for the saccharide solutions, a concentration-dependent increase in intensity was observed for the collective band, but only a small change was observed in the intensity of the band at ∼3374 cm^–1^.

**Figure 1:**
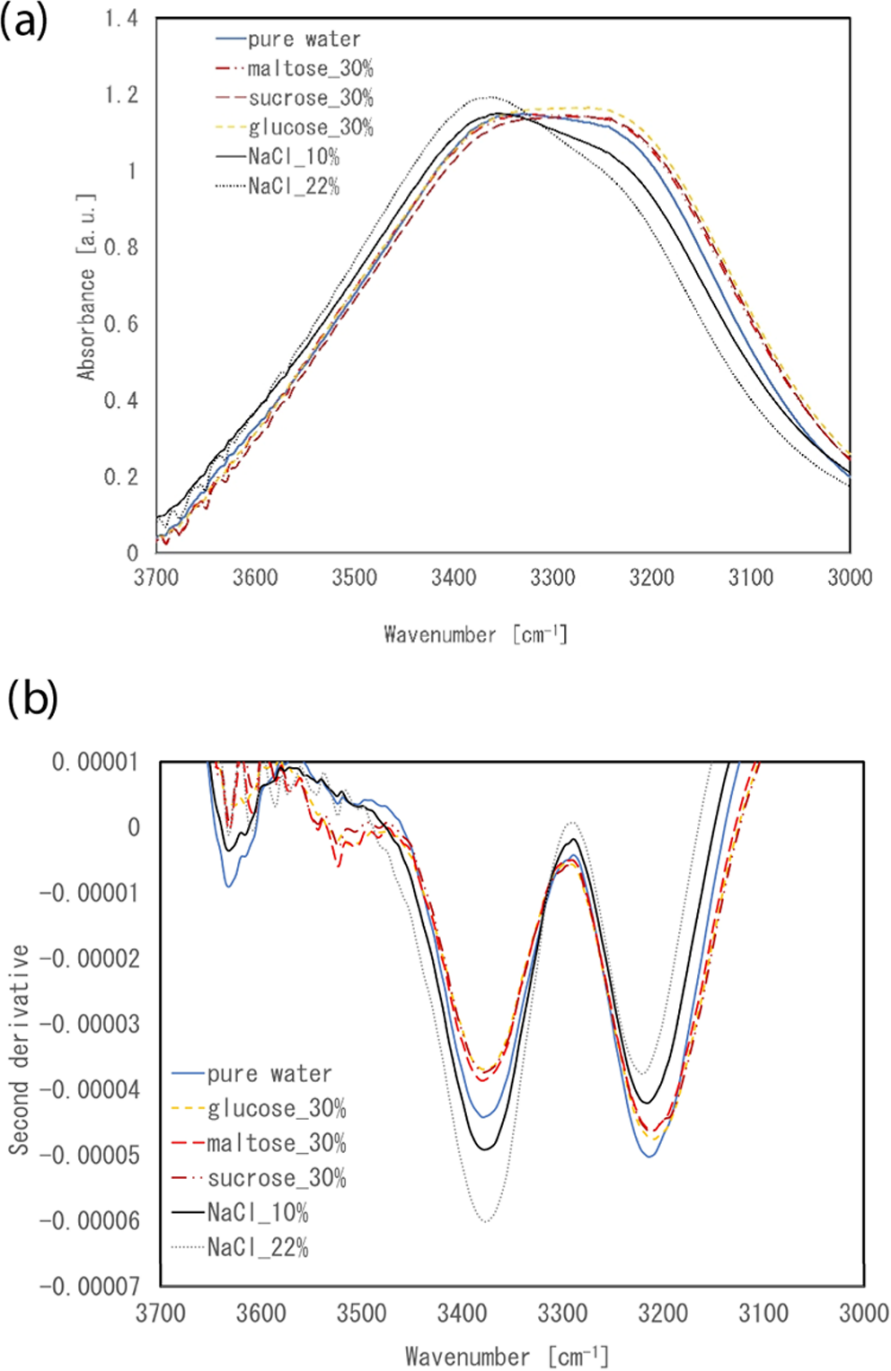
(a) IR spectra and (b) their second-derivative spectra in the 3700–3000 cm^–1^ region of three kinds of saccharide solutions, NaCl solutions, and pure water.

The IR/FIR spectra in the 1000–100 cm^–1^ region for the NaCl and saccharide solutions are shown in Figure S1. For the NaCl solution, a broad band was observed in the 800–200 cm^–1^ region. While the peak of the band occurred at around 550 cm^–1^ for the 10% NaCl solution, the peak appeared at a lower wavenumber at around 420 cm^–1^ for the 22% NaCl solution. Additionally, an isosbestic point was observed in the 500–400 cm^–1^ region for all NaCl concentrations.

Similar to a previous study,^21^ in NaCl solutions, we further analyzed the IR/FIR spectra of the NaCl and saccharide solutions in the 1000–100 cm^–1^ region via a combination of Gaussian and Lorentz functions using the curve-fitting program in Spectral Manager. The results for the NaCl solutions are shown in Figure S2 (note that this figure is reproduced from our previous study).^21^ Bands were identified in the 800–200 cm^–1^ region, specifically around 673 and 403 cm^–1^. While the intensity of the band at 673 cm^–1^ decreased with an increasing NaCl concentration, that of the band at 403 cm^–1^ increased. For the NaCl solution, the bands at 673 and 403 cm^–1^ reflect the ordered and distorted structures of the hydrogen bonds of water, respectively. Thus, the change in the intensity of the band at 673 cm^–1^ may correspond to the band at 3210 cm^–1^. As the IR/FIR spectra in the 1000–100 cm^–1^ region reflect more cooperative vibrations of the molecules than that in the 4000–3000 cm^–1^ region, the band reflects the structure of water with more macro area than the collective band.

All spectra exhibit broad bands around 600 cm^–1^ in the 800–200 cm^–1^ region, irrespective of saccharide species or concentration. Bands appearing at 990 and 928 cm^–1^ were usually assigned to the COH bending modes of the saccharides.^25,26^ Additionally, the band intensity in the 800–200 cm^–1^ increased with the saccharide concentration, and slight changes of the band shape occurred in comparison with that of pure water, possibly due to skeletal vibration of the saccharides. The results of curve fitting for the saccharide solutions are shown in Figure 2. Two bands were identified in the 600–300 cm^–1^ and 800–600 cm^–1^ for all saccharide solutions. Note that the band around 150 cm^–1^ may be due to the hydrogen bond stretch mode through a translational displacement of water molecules.^27^ Although understanding the exact behavior of this band is also an important concern, in this study, we focused on two bands around the 800–200 cm^–1^ region. The contribution of the band in the 600–300 cm^–1^ range could be higher than that of the band in the salt solution. Since two bands were identified in this region, the two-state model analysis of water may be applicable to sugar solutions. However, the spectra of saccharide solutions did not exhibit equal absorption points as in the case of salt solutions. In addition, as mentioned previously, the bands derived from skeletal vibrations were considered to be present within the bands. Therefore, even in the case of saccharide solutions, the bands extracted by the NaCl solution, which were clearly separated into two states, were considered reasonable to evaluate the states of water.

**Figure 2:**
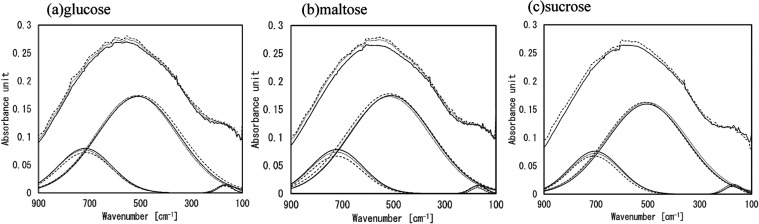
Calculated IR/FIR spectra by curve-fitting procedure and observed IR/FIR spectra in the 900–100 cm^–1^ region of (a) glucose, (b) maltose, and (c) sucrose. Note that the solid, dotted, and broken lines indicate the 10, 20, and 30% concentration of saccharide solutions, respectively.

To investigate changes caused by the additives on the tetrahedral structures formed by the hydrogen bond network, the relationships between the relative intensities of the bands at 3210 and 3374 cm^–1^ were evaluated for the NaCl and saccharide solutions (Figure 3). For standardization, the band intensities were compared according to their individual ratios to the sum of the two band intensities: A_3210_/A_3210_ + A_3374_ and A_3374_/A_3210_ + A_3374_. For both types of solutions, linear relationships were observed between the values of A_3210_/A_3210_ + A_3374_ and A_3374_/A_3210_ + A_3374_. For the NaCl solution, the value of A_3374_/A_3210_ + A_3374_ was higher than that obtained from pure water, and the value of A_3210_/A_3210_ + A_3374_ decreased linearly with increasing NaCl concentration. However, for the saccharide solutions, the value of A_3210_/A_3210_ + A_3374_ increased with saccharide concentration and was lower than that of pure water. Interestingly, the relationship between A_3210_/A_3210_ + A_3374_ and A_3374_/A_3210_ + A_3374_ for the NaCl solutions, saccharide solutions, and pure water described the same straight line.

**Figure 3:**
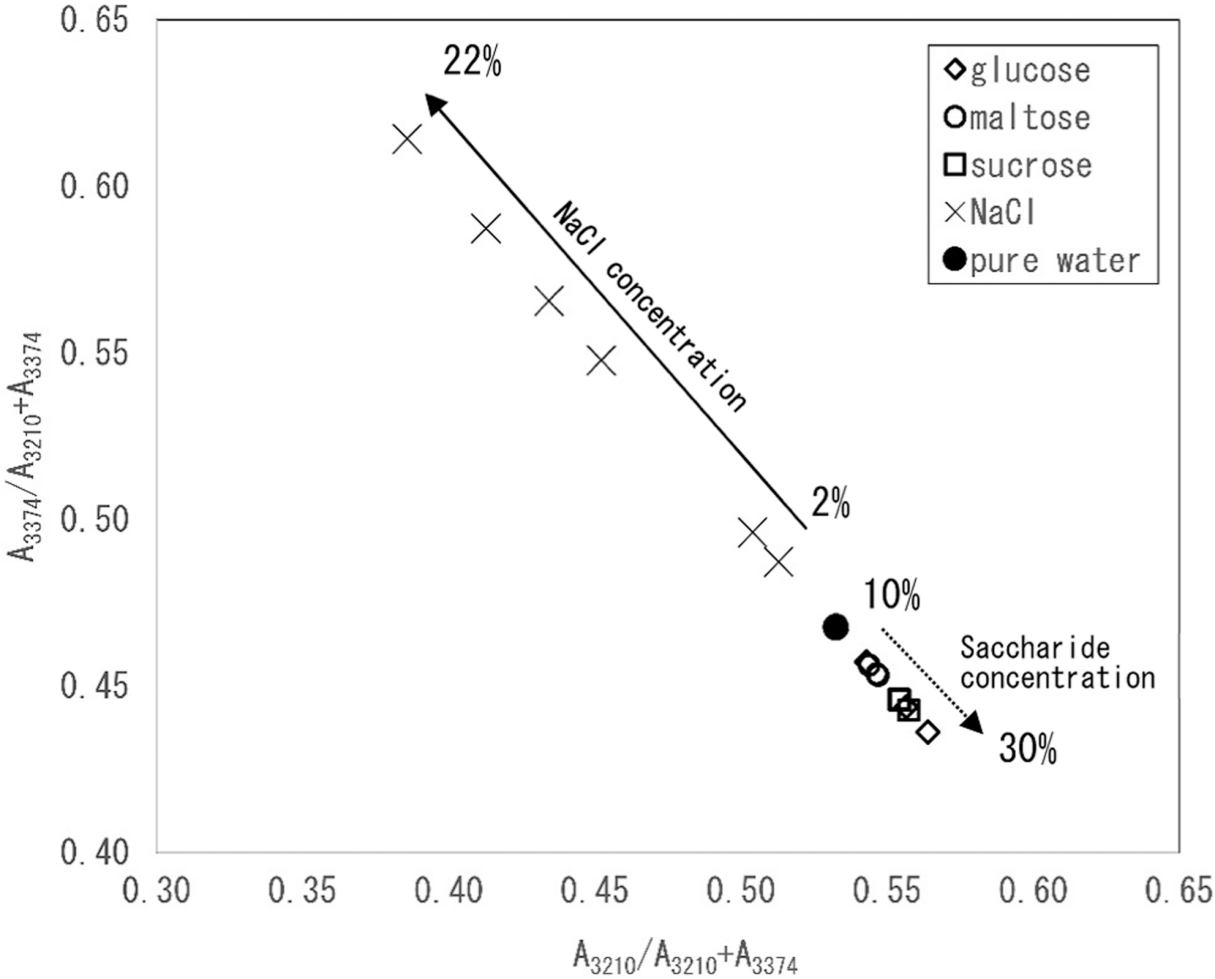
Relationship between the intensities of the 3210 and 3374 cm^–1^ bands of saccharide solutions, NaCl solutions, and pure water. Note that each value was standardized by the sum of the band intensities at 3210 and 3374 cm^–1^.

To evaluate the state of water, we proposed a ratio index, A_high_/A_low_, using two bands identified at high and low wavenumbers in the 800–200 cm^–1^ region. A_high_ and A_low_ correspond to the intensity of these bands, reflecting the ordered and distorted water conditions, respectively. As mentioned above, the wavenumbers at approximately 673 and 403 cm^–1^ identified from the NaCl solution were used in this study as the specific wavenumbers that best represent the two states of water. Furthermore, the ratio of the intensity of the collective band at 3210 cm^–1^ to that of the band at 3374 cm^–1^, that is, A_3210_/A_3374_, was considered as a valid indicator to evaluate the water states formed by the tetrahedral hydrogen-bonding structures in the NaCl and saccharide solutions. Plots of the intensity ratios A_3210_/A_3374_ and A_high_/A_low_ versus saccharide concentration are shown in Figure 4a,4b, respectively; notably, both A_3210_/A_3374_ and A_high_/A_low_ increased in proportion to the saccharide concentration.

**Figure 4:**
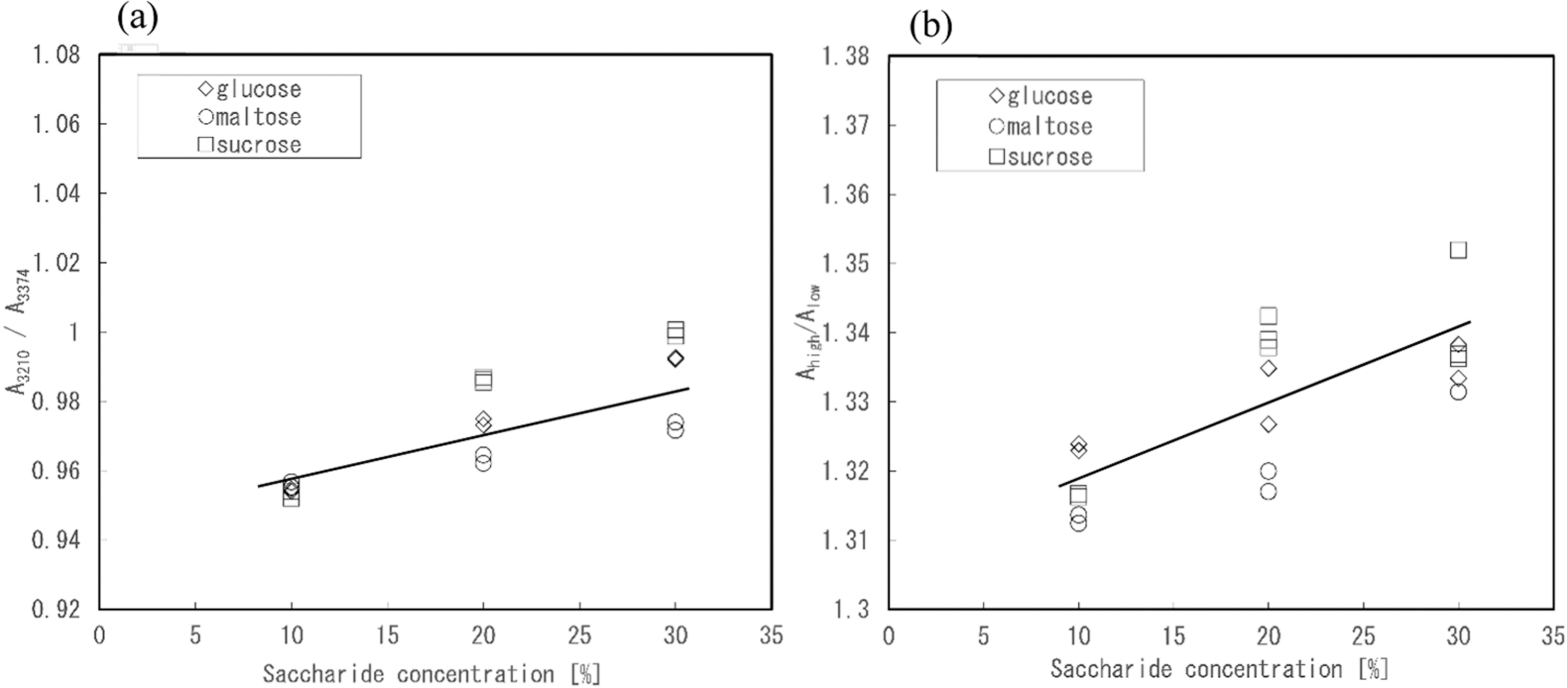
Saccharide concentration-dependent changes in intensity ratio indices of (a) A_3210_/A_3374_ using a collective band at 3210 cm^–1^ and a band at 3374 cm^–1^ and (b) A_high_/A_low_ using two identified bands in the IR/FIR region.

Two-dimensional plots of indices A_3210_/A_3374_ and A_high_/A_low_ were constructed, as shown in Figure 5. A linear relationship between A_3210_/A_3374_ and A_high_/A_low_ was observed at an approximate A_high_/A_low_ value of 1.34. Furthermore, the A_high_/A_low_ ratio remained predominantly constant at 1.34 when the value of A_3210_/A_3374_ was greater than 0.98. The values of A_3210_/A_3374_ for the 10% saccharide solutions (indicated by the green symbols in Figure 5) were higher than those of pure water. As described above, the A_3210_/A_3374_ index, which is based on the intensities of bands at 3210 and 3347 cm^–1^, corresponds to a regular hydrogen network; thus, the index may reflect the state of a small area in the water. However, the index A_high_/A_low_, which was developed based on the intensities of peaks observed in the IR/FIR spectra, corresponds to the cooperative vibration of water molecules, and therefore, it may reflect the ordered hydrogen network over a longer range in solution than that reflected by A_3210_/A_3374_. Thus, the results obtained from the two-dimensional plots suggest that the changes in the small- and long-range structures of water were linked when the solutions had medium saccharide concentrations (between 10 and 30%), but when the saccharide concentrations are high (30%), the long-range structure may change slightly. Moreover, the values of A_3210_/A_3374_ for saccharide solutions with low saccharide concentrations (10%) were higher than that of pure water. This indicates that the addition of low concentrations of saccharides strengthened the local water structure.

**Figure 5:**
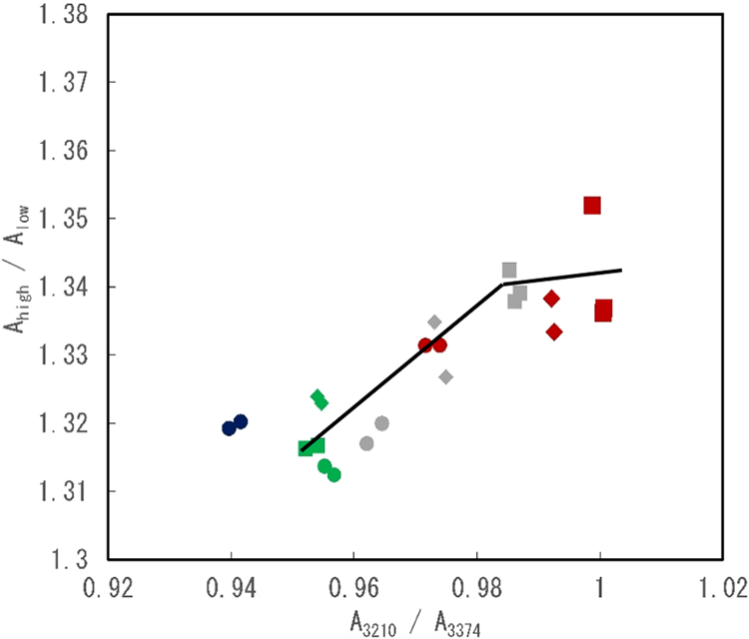
Evaluation of water state in saccharide solutions by two-dimensional plot using A_high_/A_low_ and A_3210_/A_3374_. Symbols ◇, ◯, and □ indicate the value for glucose, maltose, and sucrose, respectively. The plots of green, gray, and red colors indicate the concentration of 10, 20, and 30%, respectively. And the blue symbol indicates the value for pure water.

## Discussion

The two-state model of water macroscopically showed an ice-like tetrahedral structure, owing to the presence of the hydrogen-bonding network, wherein a part of the structure was distorted.^6^ Previous reports have shown that ions strongly distort the tetrahedral structure of water.^28,29^ For instance, studying the IR spectra of water nanodrops comprising ions in the 3800–2800 cm^–1^ region showed that the ion-induced effect on the water structure propagated over a significant distance of more than 1 nm from the ion, encompassing 250 water molecules.^29^ Thus, IR/FIR spectra in the 1000–100 cm^–1^ region, which reflect the cooperative motion of a number of water molecules,^20^ indicated distortions in the hydrogen bonds by ion addition.

The indices A_high_/A_low_ and A_3210_/A_3374_ of the saccharide solutions increased with an increasing saccharide concentration, suggesting that the addition of saccharides promoted the ordered structure of water in the solutions. This is in agreement with a previous study that reported the enhancement effect of glucose on the tetrahedral hydrogen bond network of water.^30^ Meanwhile, previous studies have reported that the action of monosaccharides and disaccharides on the organization of water is modest.^31,32^ Suzuki reported that saccharides exhibit two types of hydrogen bonding: intermolecular bonding and hydrogen bonding with hydrogen bond acceptors. Bonds with hydrogen bond acceptors have weak interactions with water, suggesting that the hydroxyl groups of simple saccharides are less hydrated and more incompatible with the local tetrahedral network of hydrogen bonds.^33^

In this study, we found that the band positions in the 1000–100 cm^–1^ region were almost the same for the 30% saccharide solutions as those of pure water. However, the intensities of the bands for the saccharide solutions were slightly higher than those for pure water. Hossain et al. conducted a near-IR study in the 10000–4000 cm^–1^ region, combined with two-dimensional (2D) correlation analysis, and found that at high concentrations of D-(+)-glucose in water, intermolecular hydrogen bonding between glucose molecules was possible, and water molecules formed ordered structures around the glucose.^22^ Therefore, the higher band intensities for the saccharide solutions could be attributed to D-(+)-glucose acting as a structural marker. Although no differences were observed in the index values based on the type of saccharide, the increase in the values of A_high_/A_low_ and A_3210_/A_3374_ suggested that the addition of saccharides enhanced the structuring of water in a concentration-dependent manner.

Additionally, as shown in Figure 5, the involvement of hydrogen bonds in the saccharide solutions was evaluated by using two indicators: A_high_/A_low_ and A_3210_/A_3374_. Ishikawa et al. reported that, for ionic solutions, these indicators showed linear correlation when the values were in the range of 0.6–1.05.^21^ However, in the case of the saccharide solutions, though the values of A_high_/A_low_ (calculated from IR/FIR spectra) and A_3210_/A_3374_ were correlated over a certain saccharide concentration range, the change in the values of A_high_/A_low_ was small at high concentrations. In ionic solutions, the ions greatly affect the local and long ranges of the tetrahedral hydrogen network in the bulk such that the linear relationship between A_673_/A_403_ and A_3210_/A_3374_ is maintained over the concentration in the 2–22% range. Meanwhile, in saccharide solutions, the effect on structuring was more pronounced at higher concentrations; however, the long-range effect estimated by A_high_/A_low_ was likely to be smaller than the local effect estimated by A_3210_/A_3374_. The values of A_3210_/A_3374_ were higher than that of pure water. Therefore, we speculated that the vibrational mode due to the O–H stretching of glucose also contributed to the index in the IR region (although the contribution was small), as evidenced by the ratio index. In future studies, the validity of this dimensional plot will be improved by estimating the contribution of the O–H stretching in the saccharides.

## Conclusions

In this study, we proposed an evaluation method for the state of water by the newly defined IR/FIR spectra in the 1000–100 cm^–1^ region and the IR spectra in the 4000–3000 cm^–1^ region. We developed spectral indices by calculating the ratios of the intensities of bands observed in the IR/FIR spectra to those of bands observed in the IR region to evaluate the state of water; these indices were validated by investigating aqueous solutions with saccharides.

In the 4000–3000 cm^–1^ region, bands were observed at 3210 and 3374 cm^–1^. While the appearance of these bands is well known, in this study, we found that the relationship between the intensities of the bands at 3210 and 3374 cm^–1^ could be plotted as a straight line for NaCl solutions, saccharide solutions, and pure water. Additionally, bands at 673 and 403 cm^–1^ were identified in the IR/FIR spectra of NaCl solutions with different concentrations. The two bands correspond to the ordered and distorted structures of water, respectively; thus, the ratio index A_high_/A_low_ was developed based on their intensities to quantify the ratio of the ordered structure of water. Moreover, the ratio index A_3210_/A_3374_, which was calculated from the second derivative of the bands at 3210 and 3374 cm^–1^, was used to evaluate the local state of the water. The values of both A_3210_/A_3374_ and A_high_/A_low_ increased with the saccharide concentration. This suggests that the tetrahedral structure formed by the hydrogen bonding of water becomes stronger with the addition of saccharides. Moreover, the two-dimensional plot between A_3210_/A_3374_ and A_high_/A_low_ showed a proportional relationship up to an intermediate concentration of saccharides; at higher concentrations, the change in the value of A_high_/A_low_ became small. Thus, the study demonstrated that the IR/FIR spectra, combined with IR spectra in the 4000–3000 cm^–1^, could be used to evaluate the state of water at different scales, thereby leading to an intrinsic understanding of water states in various aqueous solutions.

## Supplementary Material



## Notes

Supplementary Figure 1: IR/FIR spectra in the 1000–100 cm^‑1^ region of three kinds of saccharide solutions, 10 and 22 % NaCl solutions, and pure water, and calculated IR/FIR spectra by curve-fitting procedure. Supplementary Figure 2: Calculated IR/FIR spectra by curve-fitting procedure and observed IR/FIR spectra in the 1000–100 cm-1 region of NaCl solution with aw=0.986–0.865 (concentration of 2–22%) (DOCX)

## Abbreviations

ATR: attenuated total reflection
FIR: far-infrared
IR: infrared
