# Supplementary material for: Investigating the Water State in Saccharide Solutions by Infrared/Far-Infrared Spectra in the 1000–100 cm–1 Region Combined with Bands in the 4000–3000 cm–1 Region
Source: J Phys Chem A. 2025 Jun 10;129(28):6179–85. doi: 10.1021/acs.jpca.4c08369 (PMC12278207; doi:10.1021/acs.jpca.4c08369)
Supplement: Supplementary file 1 [file jp4c08369_si_001.docx]

**Supplementary Figure 1.** IR/FIR spectra in the 1000–100 cm^-1^ region of three kinds of saccharide solutions, 10 and 22 % NaCl solutions and pure water


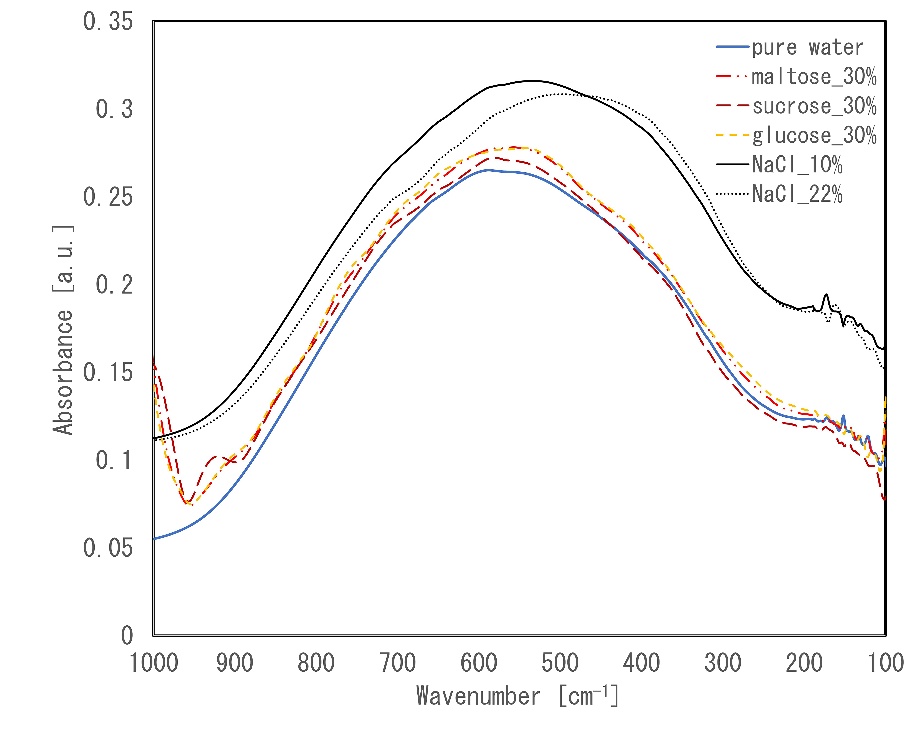

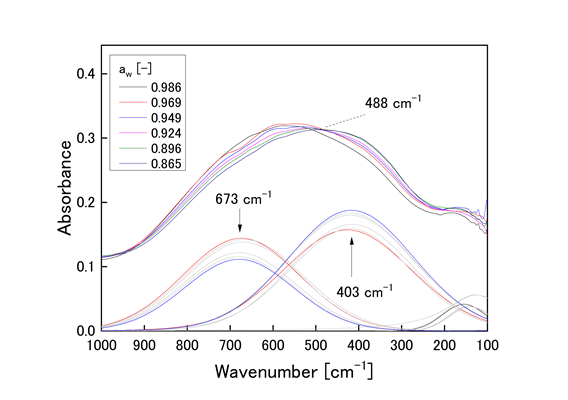


**Supplementary Figure 2** Calculated IR/FIR spectra by curve-fitting procedure and observed IR/FIR spectra in the 1000–100 cm^-1^ region of NaCl solution with a_w_=0.986-0.865 (concentration of 2-22%). Note that this figure is reproduced from Figure 3 in Ref. 21 with a modified legend. Copyright 2025, Sage Publications.
